# Impact and Correction of Analytical Positioning on Accuracy of Zircon U-Pb Dating by SIMS

**DOI:** 10.3389/fchem.2020.605646

**Published:** 2020-12-03

**Authors:** Yu Liu, Qiu-Li Li, Xiao-Xiao Ling, Guo-Qiang Tang, Jiao Li, Xian-Hua Li

**Affiliations:** ^1^State Key Laboratory of Lithospheric Evolution, Institute of Geology and Geophysics, Chinese Academy of Sciences, Beijing, China; ^2^Innovation Academy for Earth Science, Chinese Academy of Sciences, Beijing, China; ^3^College of Earth and Planetary Sciences, University of Chinese Academy of Sciences, Beijing, China

**Keywords:** SIMS, zircon, U-Pb dating, positioning effect, metamorphic overgrowth

## Abstract

Secondary ion mass spectrometry (SIMS) is one of the most important analytical tools for geochronology, especially for zircon U-Pb dating. Due to its advantages in spatial resolution and analytical precision, SIMS is the preferred option for multi-spot analyses on single zircon grain with complex structures. However, whether or how much the relative positions of multiple analytical spots on one zircon grain affect the U-Pb age accuracy is an important issue that has been neglected by most researchers. In this study, we carried out a series of investigation on the influence of relative analytical position during zircon U-Pb age analyses, using Cameca IMS 1280-HR instrument. The results demonstrated a significant influence on the second spot, with apparent U-Pb age deviation as high as around 10% especially on the left and right side with overlap in the raster area. Nevertheless, a linear correlation between a secondary ion centering parameter (DTCA-X) and age deviation in percentage terms was found, and a calibration method was established to correct this position effect. Four zircon standards (91500, M257, TEMORA-2, and Plešovice) were measured to prove the reliability of the established procedure. The original U-Pb apparent data show inconsistent deviation on four directions relative to the datum, while the final U-Pb age results is calibrated to be consistent with their recommended values, within uncertainties of ~1%. This work calls for re-examination for the previous SIMS U-Pb dating results on core-rim dating strategy, and provides a calibration protocol to correct the relative position effect.

## Introduction

Secondary ion mass spectrometry (SIMS) is widely and routinely used in U-Pb dating of zircon and other accessory minerals. It can achieve a lateral resolution of 2–30 μm and a depth resolution of 1–2 μm, which makes it the first choice for *in situ* high spatial resolution zircon U-Pb dating. By using SIMS, multiple U-Pb dating analyses on different crystal domains of single zircon grain were routinely carried out to reveal its growth processes, and then the corresponding geological evolution history (e.g., Hermann and Rubatto, 2003; Whitehouse and Platt, 2003; Zhang et al., 2005; Tang et al., 2008; Qian et al., 2013). However, when seeking higher spatial resolution for zircon U-Pb age determination, it is necessary to understand whether and how much two analytical spots with overlapped pre-analysis sputtering area or even analytical position could affect the age accuracy. Although the instrument developers were aware of this potential influence (e.g., Ickert et al., 2008), no quantitative study has been reported on this issue.

According to working principles of SIMS instrument and zircon U-Pb dating method, the sputtering, ionization, acceleration, and extraction of secondary ions are conjointly performed on the sample's surface. It means that the secondary ion yield and U/Pb element fractionation are closely related to the surface conditions, such as flatness and conductivity, of the analytical area. For SIMS analysis, zircon grains are commonly prepared in an insulated sample mount firstly (epoxy resin) and then coated with gold or carbon, to form a stable electric field between the sample surface and the extraction plate. Before each U-Pb analysis, a certain area of the conductive coating around the analytical spot is removed in pre-analysis sputtering step to reduce surface contamination. Thus, the electric field of subsequent analysis near this area will be affected by the missing conductive surroundings. This may cause a change in instrument fractionation and yield a biased U/Pb ratio if the analytical spot was placed near a sputtered area, which could be concluded to the positioning effect during zircon U-Pb dating by SIMS.

In this study, a series of experiments were designed to verify the position effect on the zircon U-Pb age during SIMS analysis. A linear correlation between a parameter of dynamic transfer contrast aperture deflector in X direction (DTCA-X) and changes in U-Pb element fractionation was observed. An effective correction protocol is proposed as a necessary supplement to the SIMS zircon U-Pb dating method to ensure the accuracy of age analysis.

## Experimental Design and Results

### Instrument Setting and Data Processing Method for Conventional SIMS Zircon U-Pb Dating

**Figure 1:**
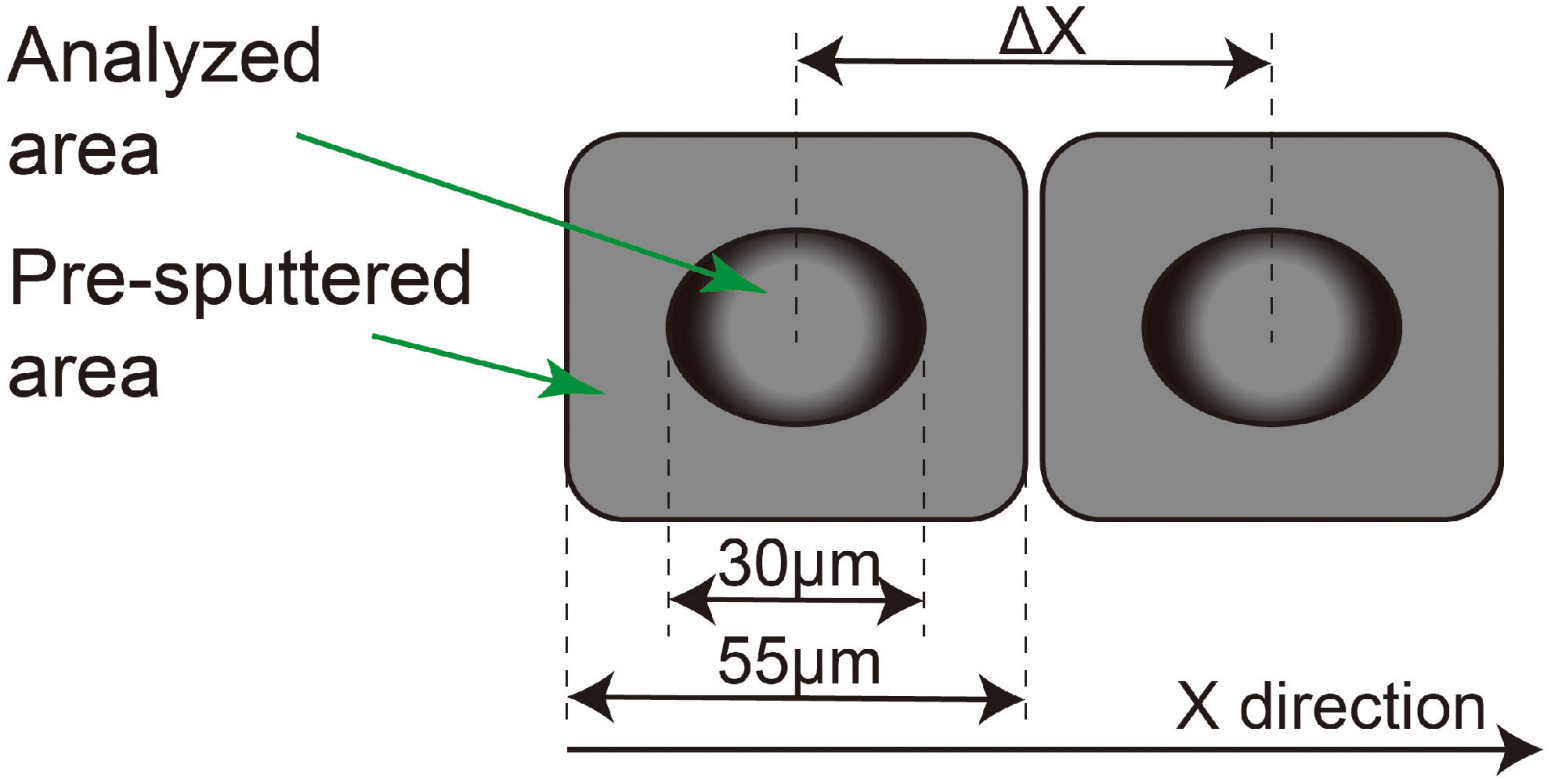
The sketch diagram of spots positioning in this article; the analysis size was 30 μm with a 25-μm pre-analysis sputtering, which makes it a 55-μm non-conducting area in the *X* direction. The Δ*X* is the distance of the two spots, center to center.

The sample mounts were cleaned and gold coated under vacuum. A conventional mono-collection mode U-Pb dating method was used in this study. Large-geometry SIMS, Cameca IMS 1280-HR, at the Institute of Geology and Geophysics, Chinese Academy of Sciences in Beijing was used in this study. The instrument settings and analytical methods are similar to that used in Li et al. (2009). The ${\text{O}}_{2}^{-}$ was selected for the primary beam and accelerated at −13 kV potential. A 6- to 10-nA beam with 30 μm × 20 μm in size was achieved by using a 200-μm PBMF (primary beam mass filter) aperture under uniform illumination mode. Before each analysis, a pre-analysis sputtering was applied for 120 s with a raster size of 25 μm × 25 μm; thus, a ~55 μm × 45 μm non-conducting area was formed (Figure 1). Then the secondary ion beam was aligned by scanning the voltage of the DTFA (dynamic transfer field aperture) and DTCA (dynamic transfer contrast aperture) deflectors, so that the beam intensity was maximized after the field aperture and entrance slit. An energy centering by scanning sample high voltage (energy bandwidth of 60 eV) and mass calibration (mass resolution power = 7,000) were performed subsequently. The data acquisition time was ~7 min, and the total analysis time was 11–12 min for each spot.

The ionization yields of Pb^+^ and U^+^ change with the analytical conditions and the U content in the sample during the SIMS analysis. Therefore, the correction of U-Pb fractionation is required. The protocol of U-Pb correction in this paper is the same as the conventional SIMS U-Pb dating method, which is based on the linear relationship between ln(^206^Pb^+^/^238^U^+^) and ln (^238^U^16^${\text{O}}_{2}^{+}$/^238^U^+^) (Compston et al., 1984; Whitehouse et al., 1997; Li et al., 2009). The external error of the calibration curve (which varies from 0.55 to 0.91% in this paper) and the internal error of each analysis point (generally <0.5%) are propagated to the final results (about 1%). Uncertainties for individual analyses in the data table and charts are reported at the 1α level, unless otherwise stated. Data reduction was carried out using the Isoplot/Ex v.4.0 program (Ludwig, 2001).

Most of the measured ^206^Pb/^204^Pb ratios are higher than 4 × 10^4^ in all sessions; thus, the effect of common Pb isotopic composition is negligible. Assuming that the common Pb are introduced during the sample preparation, the average crustal composition of Pb in the present day (^206^Pb/^204^Pb = 18.703, ^207^Pb/^204^Pb = 15.629) (Stacey and Kramers, 1975) is used for common Pb correction. Detailed error propagation of common Pb correction follows those described by Li et al. (2009).

### Experiment Design

The analysis spots were divided into two types; one type was called the datum spot, which represents the standard sample used for U-Pb fractionation correction in routine analysis. These spots were positioned on a fresh surface of a large zircon grain. The space between analysis spots was large enough (>100 μm) to ensure that there was no interference between each other.

Another type was called a testing spot in this study. To simulate the conditions of multiple analysis on a single zircon, a testing spot was positioned near a datum spot where the analysis was already done. It was placed by moving a small distance (Δ*X* or Δ*Y*) in the *X* or *Y* direction (Figure 1) from each datum spot.

The experiment was divided into three sessions. In the first session, the Δ*X* or Δ*Y* ranged from 15 to 75 μm, and the testing spots were distributed in four directions of up, down, left, and right relative to the datum spots. Every eight analyses were taken as one group, including four datum points and four testing points. For each group, four datum points were analyzed firstly and then followed by four testing points (Supplementary Figure 1). For clarity, testing points marked “right” and “up” have coordinates greater than their corresponding datum points and vice versa. There were 44 datum point analyses and 44 testing point analyses in total. In the second session, the spots spacing Δ*X* was set 30–70 μm on the left and right sides, i.e., from the zero space next to datum spot (with 30 μm beam size) to totally separate the raster area. The datum spots and the testing spots were cross-analyzed. In the first two sessions, the zircon U-Pb age standard M257 (Nasdala et al., 2008) was employed to make the estimation.

In the third session, four standard zircons, 91500 (1062.4 ± 0.4 Ma, Wiedenbeck et al., 1995), M257 (561.3 ± 0.3 Ma, Nasdala et al., 2008), TEMORA-2 (416.8 ± 1.3 Ma, Black et al., 2004), and Plešovice (337.1 ± 0.4 Ma, Sláma et al., 2008), were analyzed to establish the DTCA-X correction method. The instrument parameters and conditions were similar to that used in previous sessions. Four datum spots were selected with sufficient spacing firstly, and four testing spots were selected in up, down, left, and right of the four datum spots. The center distance between the testing spots and the datum spots was 30 μm (ΔX/ΔY). Thirty-two spots, including datum and testing spots, of 4 standard zircons made one analytical group. Five groups, i.e., a total of 160 spots analysis, were conducted in the third session. In addition, the counting time of DTCA-X centering was prolonged from 0.16 to 0.24 s/step, and the centering step was changed from 50 to 60 steps, to improve the accuracy and resolution of DTCA-X measurement.

### The Determined U-Pb Ages

#### Results of Session 1

**Figure 2:**
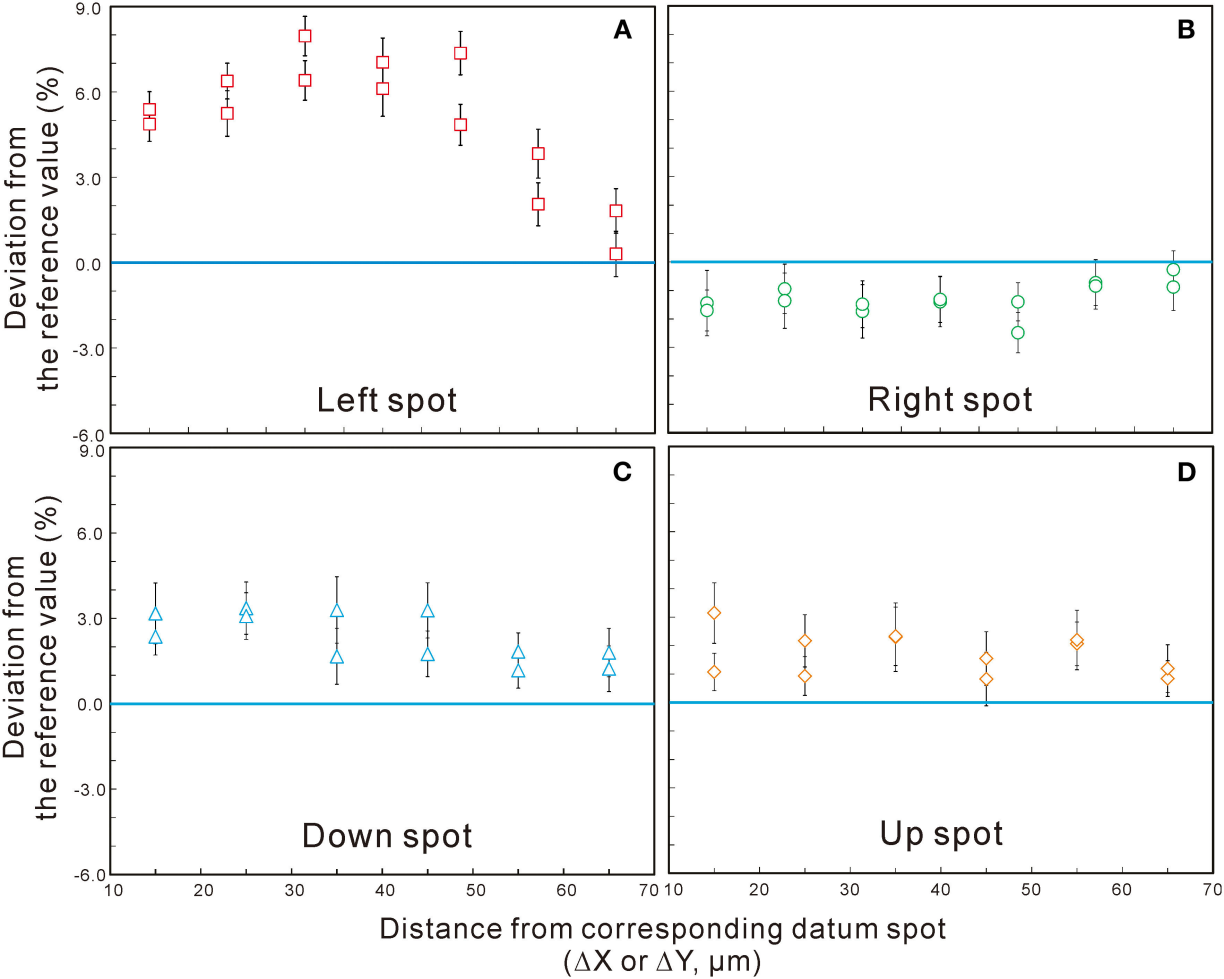
The age deviation (percent) of testing spots vs. their centering distance (μm) in four directions, **(A)** left, **(B)** right, **(C)** down, and **(D)** up, of session 1 (M257 zircon). Error bars are 1σ.

The ^238^U-^206^Pb apparent ages of datum points and their relevant testing points in session 1 are presented in Supplementary Table 1. ^238^U-^206^Pb apparent ages of 44 datum points yielded an external error of 0.61% (1RSD), which is much better than the long-term external precision of the laboratory (1.5%) (Li et al., 2010), indicating a good stability during this session. However, ^238^U-^206^Pb apparent ages for testing points in all of four directions show large variations (Figure 2), with higher values in the left, up, and down directions and lower values in the right direction. Individually, the ^238^U-^206^Pb age deviation can be up to 8% for left-offset testing points, and the dispersions in other directions are also >1%. All these deviations are far beyond the analytical uncertainty of conventional SIMS zircon U-Pb dating method (Li et al., 2009, 2010). The results of session 1 demonstrated that there is an obvious positioning effect during SIMS analysis, which has a significant impact on the ^238^U-^206^Pb apparent age results, especially on the *X* direction.

#### Results of Session 2

**Figure 3:**
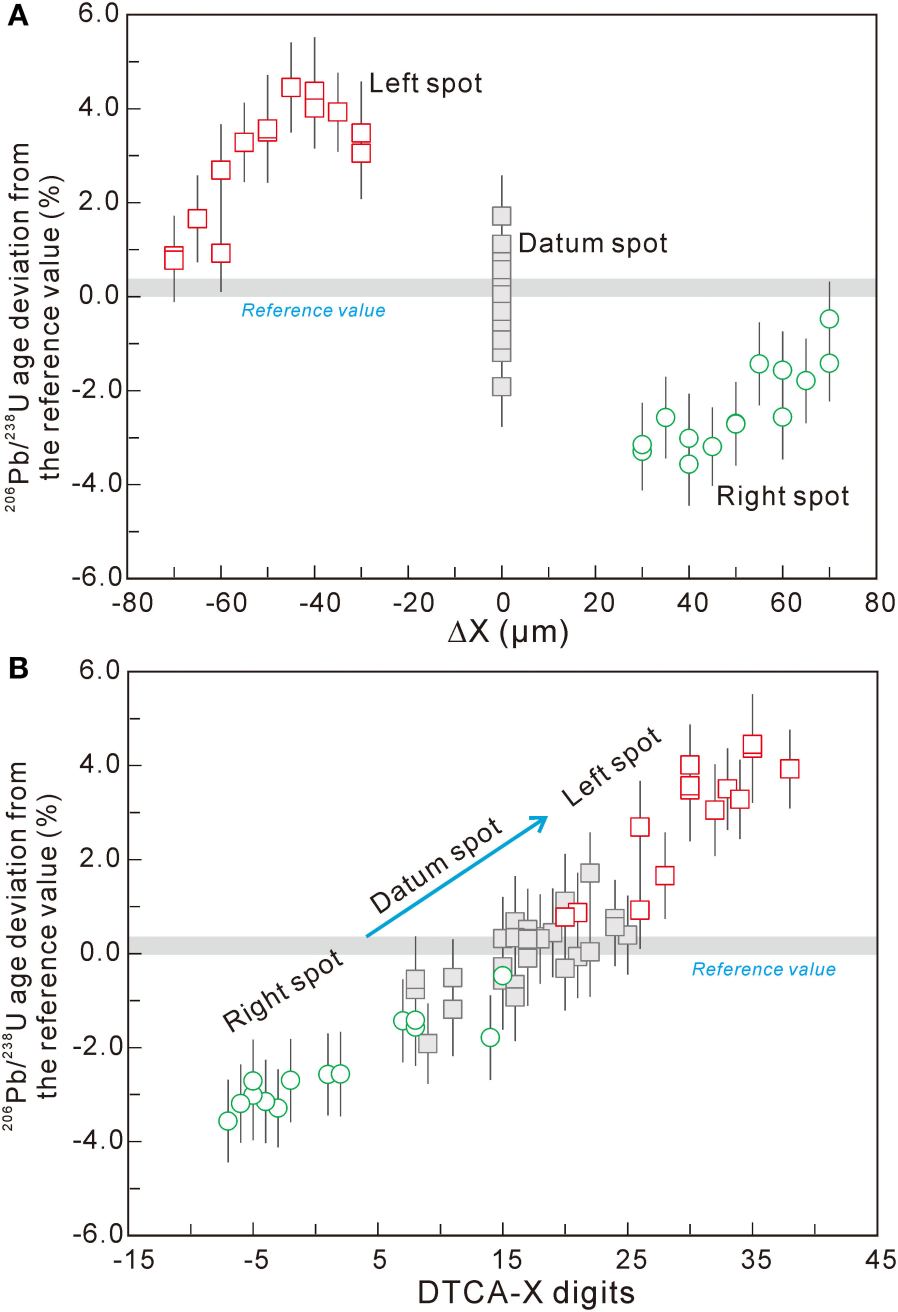
**(A)** The U-Pb age deviation from their reference value of testing spots vs. their centering distance in session 2 of M257. **(B)** The linear trend between U-Pb age deviation from their reference value and DTCA-X digit of the left and right testing spots in session 2 of M257.

The U-Pb age results of testing spots are listed in Supplementary Table 2. The overall trend of the data sets in this session is similar to that of session 1, with an external error of 0.9% (1RSD) for the datum spots and an up to 4.3% age deviation for the left side spots (Figure 3A). The U-Pb apparent ages on the left side show a deviation of +3% higher than the reference value on the spots next to datum spots. As Δ*X* increases, the U-Pb age deviation also gradually rises to as high as 4.3% at around 50 μm, where the raster areas are next to each other between two spots. Then, the U-Pb age deviation gradually decreases as ΔX increases, to reach a value consistent with the reference value range. It shows clearly that the intensive selection strategy may introduce great age bias. This should be considered during the design of the analysis.

#### Results of Session 3

**Figure 4:**
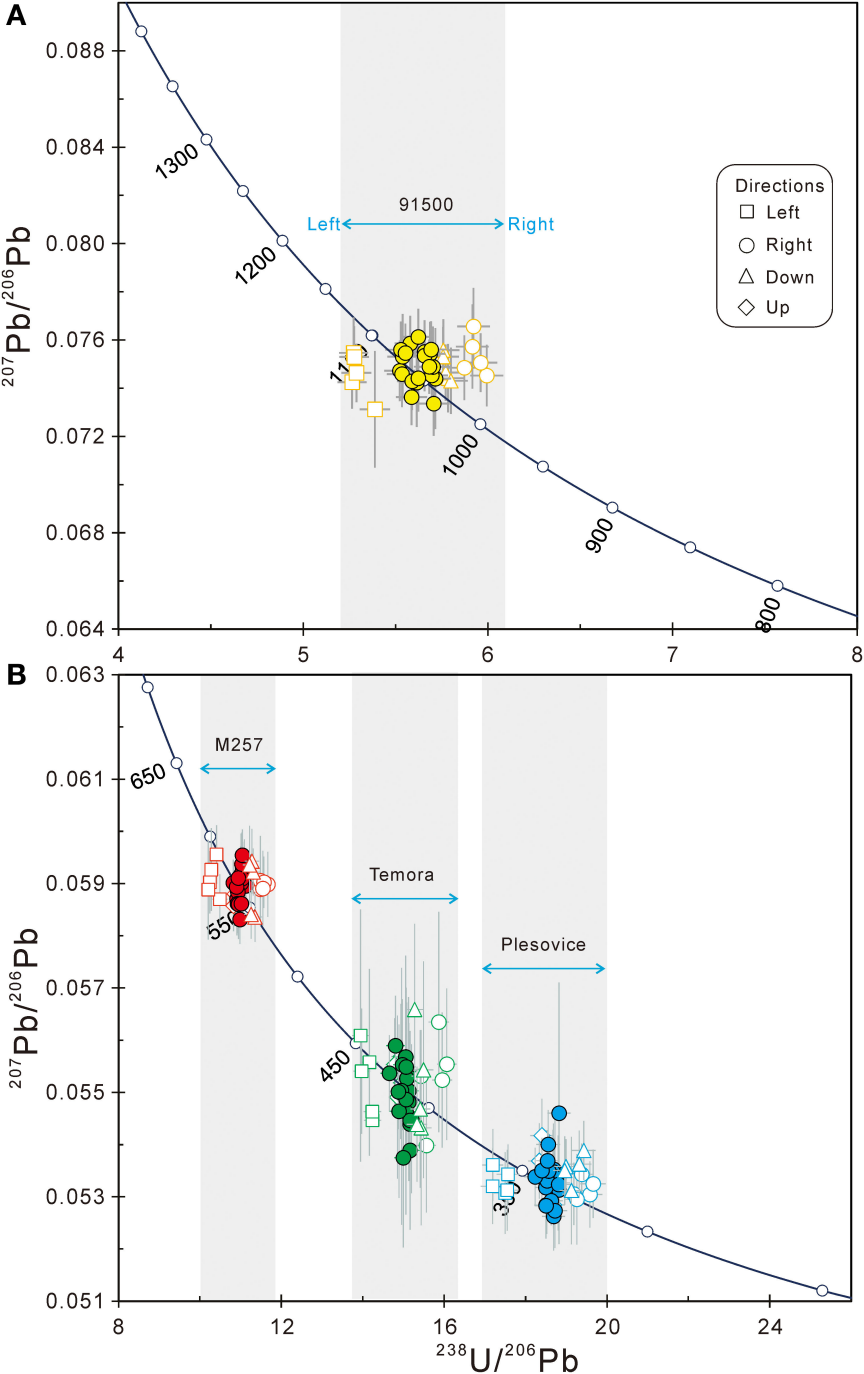
The U-Pb age of datum and testing spots of four standard zircon, **(A)** 91500, **(B)** M257, TEMORA-2, and Plešovice, in session 3. Error bars are 1σ.

After the routine U-Pb fractionation correction (datum spots of M257 were used as the reference) and common Pb correction (Li et al., 2009), the U-Pb ages of all spots in session 3 are plotted in Figure 4, and the detailed results are listed in Supplementary Table 3. The weighted average of U-Pb ages from their datum spots of the four standards 91500, M257, TEMORA-2, and Plešovice are: 1054.9 ± 5.4 Ma, 561.3 ± 1.9 Ma, 415.3 ± 1.4 Ma, and 337.4 ± 1.1 Ma (2σ, *n* = 20), respectively. The results are overlapped with their recommended values within ~1% range. The results of testing spots of the four standards scattered in a large range (Supplementary Table 3). In this session, the age deviation of the left and right testing spots are up to 10%, while the up and down spots are around 3%.

## Correlation Between DTCA-X Parameter and U-Pb Age

**Figure 5:**
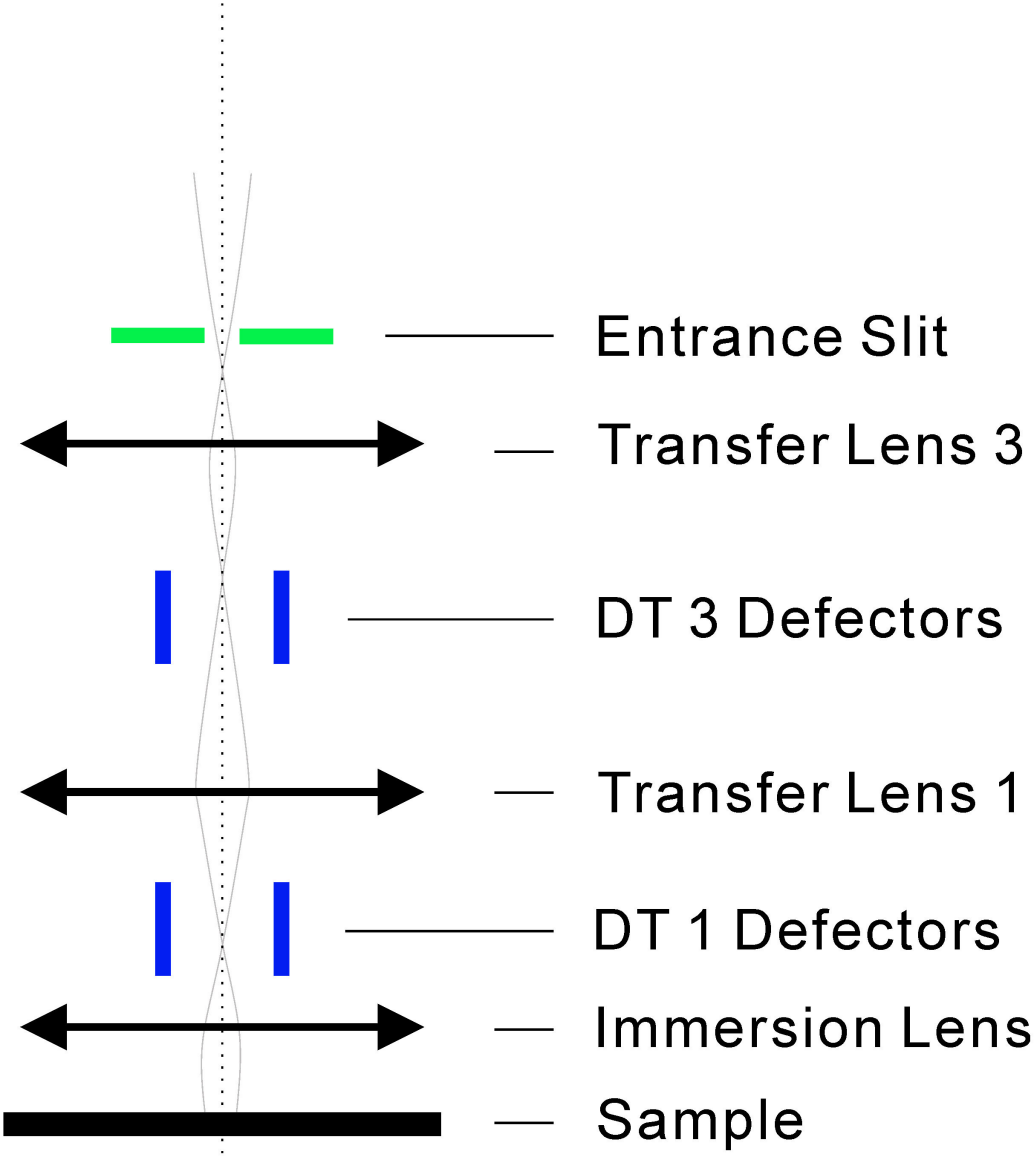
The schematic diagram of DTCA deflectors. The DT1 and DT3 work together to guide the secondary ion beam through the entrance slit with maximum intensity. DT represents transfer deflector.

Among many instrument parameters, this study found that there was a strong correlation between the instrument parameter of DTCA-X and the age of U-Pb. The DTCA-X parameter of the Cameca IMS-1280 SIMS and later version represents a set of deflectors, which is used to center the secondary ion beam at the entrance slit (Figure 5). The working principle of DTCA-X is to change the trajectory of the secondary ion beam by scanning the voltage of the DTCA-X deflectors to maximize the intensity after the entrance slit. It helps to maintain the consistency of the analytical conditions under different sample surface conditions (Liu et al., 2019).

The digital value of DTCA-X was recorded for each spot. The obtained U-Pb age deviation from their reference values in percent of session 2 was plotted against the corresponding DTCA-X value in Figure 3B. The U-Pb apparent ages of this group have a strong positive correlation with its corresponding DTCA-X, providing us with a potential correction method to suppress the positioning effect.

The importance of secondary ion beam centering has been stressed by instrument developers and users, including both SHRIMP and Cameca instruments (Ireland and Williams, 2003; Schuhmacher et al., 2004; Ickert et al., 2008; Ireland et al., 2008; Peres et al., 2008; Whitehouse and Nemchin, 2009). We also found that this parameter can be used to improve the accuracy of Si-O isotope analysis in the previous work (Liu et al., 2019). In order to find a proper method of U-Pb age correction to overcome this positioning effect, the data set of session 3 was used to study the role of DTCA-X in U-Pb correction.

### U-Pb Age Correction Using DTCA-X

**Figure 6:**
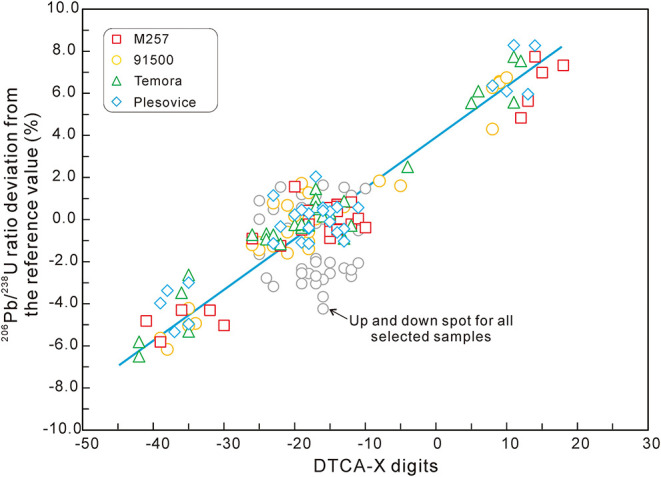
The linear trend between the relative deviation of U-Pb age and DTCA-X digit of four zircon standards in session 3.

The correlation between the relative deviation of the U/Pb ratio and the DTCA-X parameters in session 3 is shown in Figure 6. The colored spots are the ones of datum spots and their left and right testing spots. It shows that all the four zircon standards have a strong linear trend with very similar slopes. The up and down testing spots (gray circles) do not show any trend in this diagram. For example, the linear correlation coefficient *R*^2^ obtained for M257 zircon is only 0.79 for all the spots, but the value can reach 0.93 without the up and down testing spots.

### Protocol of U-Pb Age Correction Using DTCA-X

The data of session 3 (Supplementary Table 3) is taken as an example to specifically explain the correction method (left and right testing spots) for the U-Pb age deviation. M257 is used as the standard, and the remaining samples 91500, TEMORA-2, and Plešovice were treated as unknown samples. The following are the correction steps:

Calibrate the U-Pb fractionation against M257 using the routing method (Li et al., 2009).

Calculate the relative deviation of Pb/U ratio of M257 (the “Pb/U ratio deviation %” column in Supplementary Table 3); the average ^206^Pb/^238^U ratio of datum M257 spots (0.0910) were used as the reference value.

Testing spots in the left and right directions as well as the datum spots of M257 were linearly fitted using “DTCA-X” and “^206^Pb/^238^U ratio relative deviation %” data. The linear equation *y* = 0.2162*x* + 3.2896 was obtained in this example, and the slope 0.2162 was used as the correcting slope for all the other samples, expressed as *a*.

The average value of DTCA-X of the M257 spots in step 3 (−13.93, in this example) is used as the reference value of DTCA-X, expressed as *A*.

The Pb/U ratio (“^206^Pb/^238^U” in Supplementary Table 3) was expressed as *R1* and the corresponding DTCA-X value was expressed as *C*; thus, the Pb/U ratio can be corrected by using the formula:

(1) $$\begin{array}{r} R_{DTCA-X}=R1\times \left[1-\left(C-A\right)\times \;a/100\right] \end{array}$$

*R*_DTCA−X_ is the corrected Pb/U ratio shown in Supplementary Table 3 (up and down testing spots were not corrected using this method). Thus, all the U/Pb ratios are corrected based on the difference of their corresponding DTCA-X values (*C*) with average DTCA-X value of M257 (*A*).

The *R*_DTCA−X_ external error (except for the up and down testing spots) of M257 (0.91%, 1RSD, in this example) was calculated propagated to the final age errors.

## Results of DTCA-X Correction

**Figure 7:**
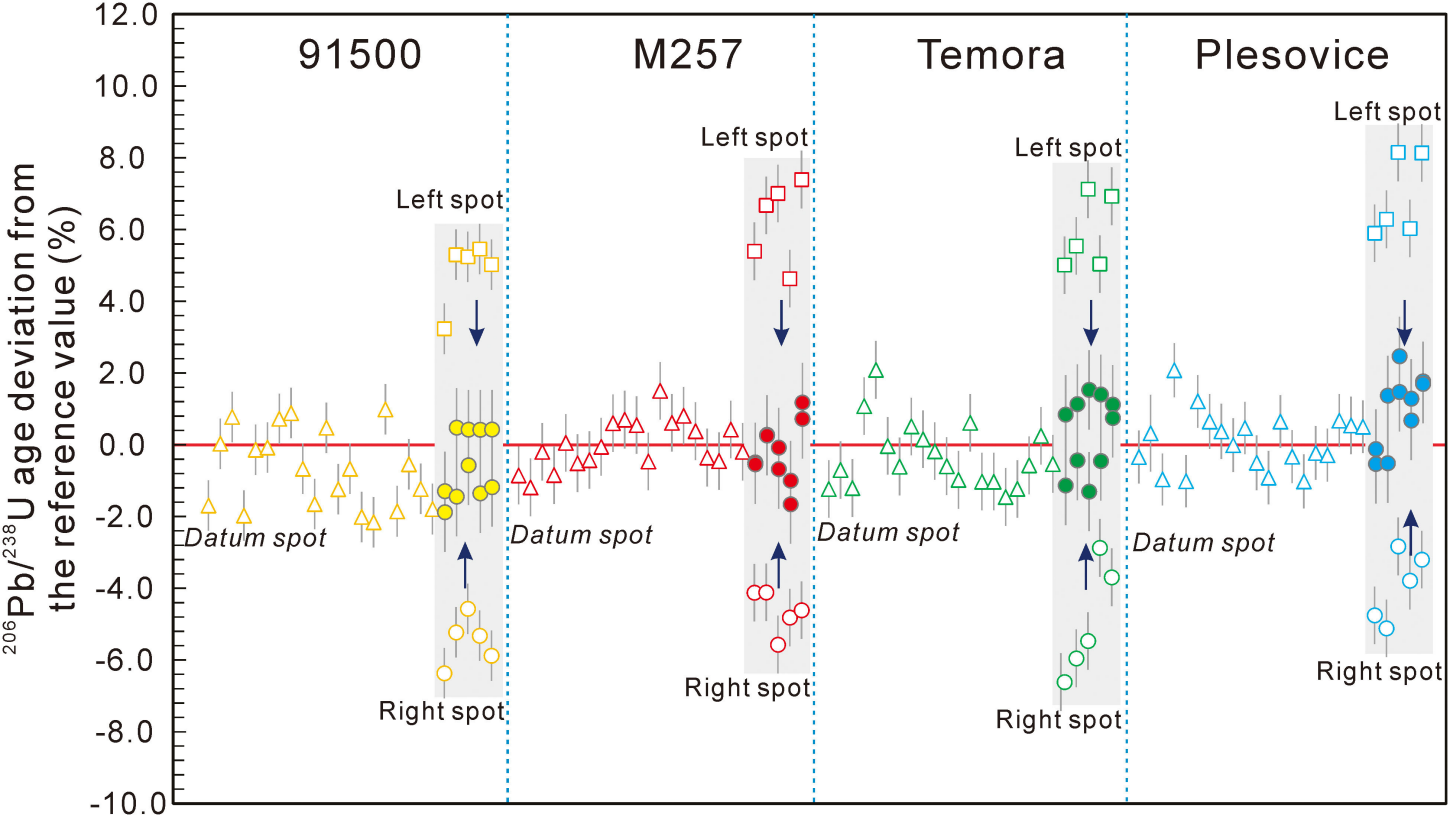
The U-Pb age deviation of datum and testing spots on the left and right side of four standard zircons. The hollow and solid points represent the data before and after the DTCA-X correction, respectively.

The data before and after the DTCA-X correction in session 3 are listed in Supplementary Table 3 and plotted in Figure 7 (except the up and down testing spots). After the DTCA-X correction, the U-Pb age distribution ranges of all samples are reduced significantly. The weighted average U-Pb ages (excluding the up and down testing spots) of the four standard 91500, M257, TEMORA-2, and Plešovice are 1062.0 ± 4.2 Ma, 562.2 ± 2.4 Ma, 418.7 ± 1.8 Ma, and 339.8 ± 1.4 Ma, respectively, after DTCA-X correction. The results are consistent with their recommended values within an error of ~1%, and the external reproducibility of each sample (1 RSD) is also ~1%.

## Discussion

### Positioning Effect of SIMS Zircon U-Pb Dating

A series of experiment shows that multiple analyses within a small range may cause a large deviation in age results. Therefore, attention must be paid not to set multiple analyses in a small area if no further calibration is performed. More importantly, the degree of the deviation varies with the instrument conditions and the tuning details. The deviation does not show a constant trend with the offset distance (Δ*X* or Δ*Y*) within a same session. Thus, it is difficult to find a straightforward method for the correction, unless the DTCA-X parameter was used.

In general, when Δ*X* and Δ*Y* were close to or >75 and 65 μm, respectively, the measured result was consistent with the reference value within the error. In another word, the gold coating with a width of more than 15 μm should be kept around the analysis spot for routine applications.

### The Possible Causes of U-Pb Fractionation Changing

Firstly, the charge accumulation on the previous analysis spots may have some effect on the changing of the U-Pb fractionation of the following analytical spots. However, according to the experiments design, the time intervals of datum spots (grouped in 4) and their corresponding testing spots are more than 50 min. This time interval is long enough to release the accumulated charge. So, this factor is not likely the reason of the phenomenon in this study.

Secondly, the parasitic magnetic field in the environment combined by geomagnetism and other magnetic equipment such as the steel frame of the instrument and the ion pump will interfere with the ion trajectory. Mass fractionation caused by the parasitic magnetic fields is generally in a relatively constant level, but it can result in variable isotope and elemental fractionation when combined with different surface conditions of the sample (Liu et al., 2019). For this study, the fractionation changes of U and Pb are likely due to the difference in the conductive conditions of the sample surface (datum spot and testing spots).

Such mass fractionation generally occurs at the slit of the ion optical path, for instance, the entrance slit, the field aperture, and the energy slit. The Cameca IMS-1280/HR SIMS is equipped with several sets of deflector plate (for example, DTCA, DTFA, etc.) to change the direction of secondary ion trajectory. By adjusting voltages of the deflectors, the intensity of secondary ion beam can be maximized at the entrance slit and the field aperture of each analysis spot. Among these centering parameters, the DTCA-X (related to entrance slit) was found to have a strong relationship with U/Pb fractionation change in the left and right side. Meanwhile, neither the results of the energy scan (sample HV offset) nor DTFA digit (related to field aperture) has obvious correlation with this fractionation change. It indicates that the fractionation change is more likely to occur at the entrance slit. However, no such trend was found for the up and down spots, indicating that the reason is not yet studied. In different sessions, the data of the up and down testing spots may even deviate in different directions. For example, the data of the up and down spots in session 1 is higher than the reference value, while in session 3, it is lower. More parameters need to be monitored in the future work to reveal the cause of the fractionation in these two directions.

**Figure 8:**
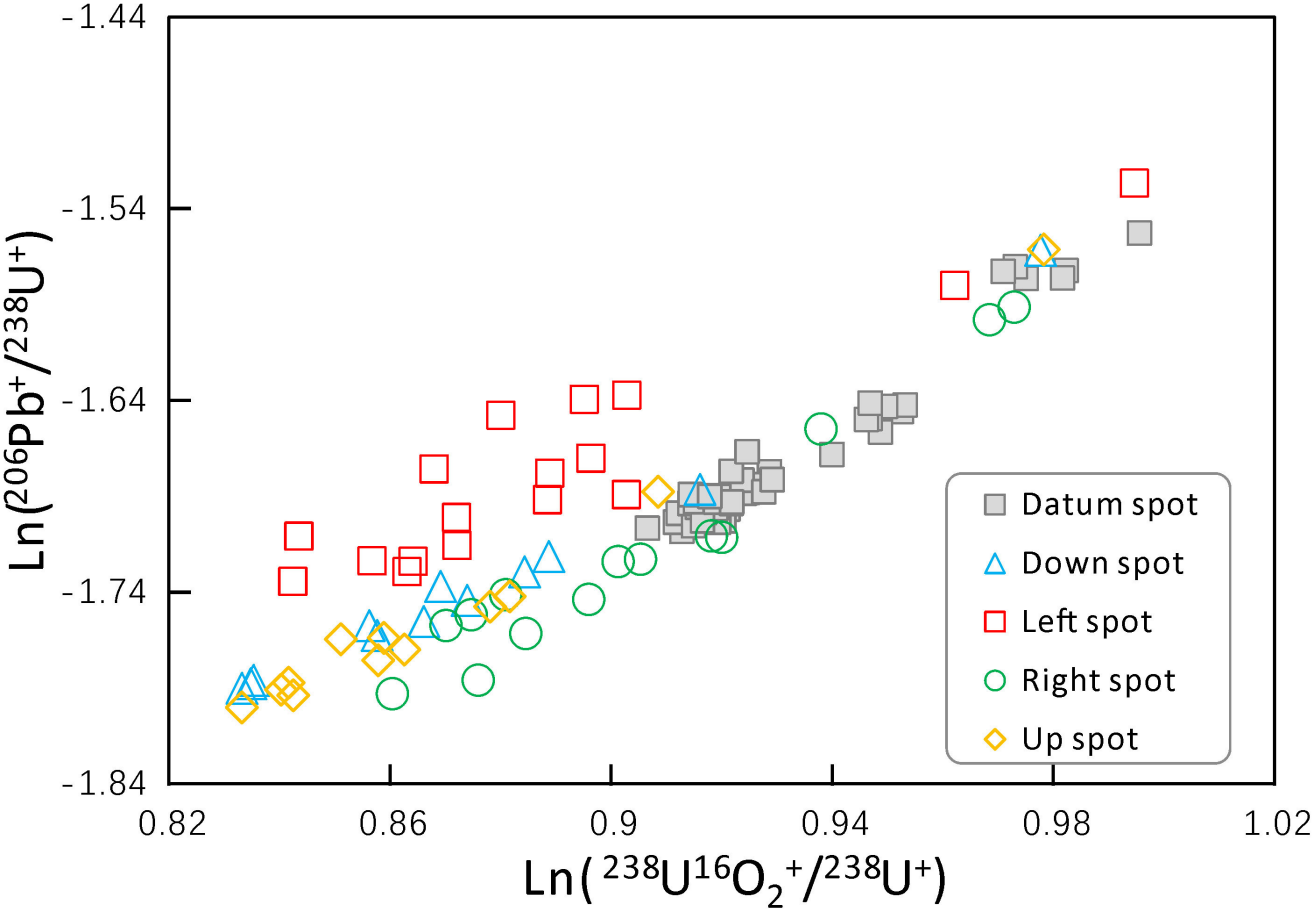
The relationship of measured Ln(^238^U^16^${\text{O}}_{2}^{+}$/^238^U^+^) and Ln(^206^Pb^+^/^238^U^+^) of session 1 data. Most of the testing spots are deviated from the calibration line formed by the datum spots. The most deviated “left” spots show that the change of Pb/U ratio may be the main cause of age deviation.

Additionally, the two main ratios that determine the U-Pb age, Pb^+^/U^+^ and U_2_O^+^/U^+^, are both influenced by the positioning effect. In Figure 8, these two ratios of session 1 data are plotted. Most of the testing spots are located in the lower left part of the chart, which means the positioning effect would cause the decrease of both ratios. From the data points of the most deviated “left” testing spots, it is obvious that the drift of the Pb^+^/U^+^ ratio is the main cause of the age deviation.

It is worth noting that although the U-Pb apparent age was greatly affected by the positioning effect, the Pb-Pb age is almost unaffected, indicating that the isotope fractionation changes under this condition are negligible. This may be related to the mass difference. The relative difference between the ^206^Pb and ^207^Pb isotope masses determined for the Pb-Pb age calculation is ~0.5%, while the difference between the masses used to calculate the U-Pb age can be >20%. Or it may be related to the difference in ionization properties between different element species. Therefore, the Pb-Pb age is more reliable in the case of intensive analysis.

### The Feasibility of Correction for up and Down Testing Spots

The DTCA-X correction method is not applicable for the up and down testing spots. However, we found that within one session, the variation range of these testing spots is relatively constant. For instance, although the center distance (Δ*Y*) varied over a large range (15–65 μm), the standard deviation of the age data (up and down spots) in the first section is about 1% (Supplementary Table 1). In this case, the average deviation of the standard sample can be used to correct the unknown samples. If we apply this correction method to the data of session 3 (M257 as standard), the average deviation of up and down spots in each sample, 91500, TEMORA-2, and Plešovice, is reduced to −0.7, −0.14, and −0.1%, respectively. The corrected data are consistent with the recommended values within errors, and the accuracy is comparable with the conventional U-Pb dating accuracy of 1% level (Supplementary Table 3, up and down spots).

## Conclusions

1. A series of positioning effect tests for SIMS zircon U-Pb dating were investigated in this study. Significant U-Pb age deviations were observed and varied with different spacing in four directions (left, right, up, and down) away from the datum points. The gold coating with a width of more than 15 μm should be kept between different analysis spots.
2. The U-Pb age deviation of the lateral offset spots could reach 13%. The lateral effect can be calibrated based on an observed correlation between DTCA-X and age deviations. The corrected results are consistent with the recommended values within 1%.
3. The U-Pb age deviation of the up and down spots can be corrected by the relative constant deviation value determined by the concurrent analytical zircon standards.

## Data Availability Statement

The original contributions presented in the study are included in the article/Supplementary Material, further inquiries can be directed to the corresponding author.

## Author Contributions

YL found the correction method used in this study and wrote the draft of the article. Q-LL helped in experiment layout and data processing. X-XL was the first one who found two spots which placed too close would have positioning effect. G-QT helped tuning the instrument. JL helped preparing the samples. X-HL provided the instrument using experience of SHRIMP and helped destining the experiments. All authors contributed to the article and approved the submitted version.

## Conflict of Interest

The authors declare that the research was conducted in the absence of any commercial or financial relationships that could be construed as a potential conflict of interest.
