# Supplementary figures and images for: Impact and Correction of Analytical Positioning on Accuracy of Zircon U-Pb Dating by SIMS
Source: Front Chem. 2020 Dec 3;8:605646. doi: 10.3389/fchem.2020.605646 (PMC7744676; doi:10.3389/fchem.2020.605646)

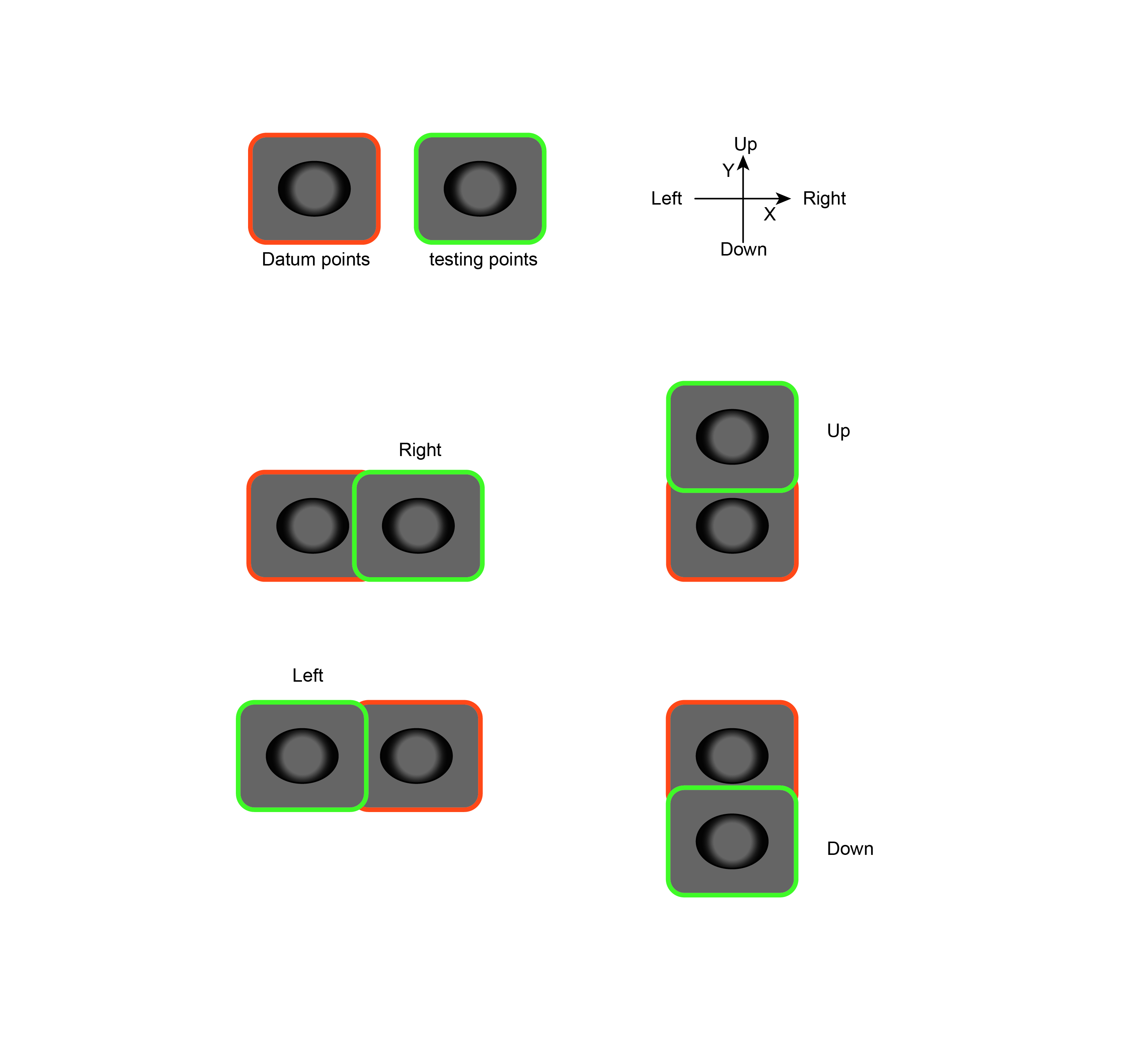

Supplement: Supplementary file 1 [file Image_1.TIF]
